# Strength–Toughness–Wear Coupling Mechanisms of Low-Carbon Martensitic Wear-Resistant Steel Enabled by Ti/Nb Microalloying-Driven Carbide Precipitation and Synergistic Regulation of Tempered Microstructures

**DOI:** 10.3390/ma19102043

**Published:** 2026-05-13

**Authors:** Qunjiao Wang, Jiangong Zhou, Dapeng Wang, Jun Miao, Chunming Liu

**Affiliations:** 1 Key Laboratory of Electromagnetic Processing of Materials (Ministry of Education), Northeastern University, Shenyang 110819, China; 2 School of Materials Science and Engineering, Northeastern University, Shenyang 110819, China; zhoujiangong_off@163.com (J.Z.); 15716106877@163.com (D.W.); cmliu@mail.neu.edu.cn (C.L.); 3 Benxi Steel Group Co., Ltd., Benxi 117000, China; miaojun_off@163.com

**Keywords:** low-carbon martensitic steel, MC-type carbide precipitation, Ti/Nb microalloying, dry-sliding wear, fatigue-induced spalling, third-body effect

## Abstract

The effects of Ti/Nb microalloying-induced MC-type carbide precipitation and tempered microstructure evolution on the dry-sliding wear behavior of low-carbon martensitic wear-resistant steels were systematically investigated. Three experimental steels with different microalloying strategies (0.04Ti, 0.1Ti, and 0.04Ti/Nb) were subjected to quenching and subsequent tempering. Microstructural features, carbide characteristics, and mechanical properties were characterized using optical microscopy (OM), scanning electron microscopy (SEM), X-ray diffraction (XRD), transmission electron microscopy (TEM), tensile testing, and impact testing, while wear performance was evaluated by pin-on-disk tests under dry-sliding conditions. The results indicate that wear resistance is governed by the combined effects of tempered martensite stability and MC-type carbide precipitation. Low-temperature tempering effectively reduces the wear mass loss of Ti-containing steels by enhancing their resistance to abrasive shear deformation while maintaining sufficient toughness. In contrast, the Nb-containing steel exhibits a stage-dependent wear response associated with the formation and destabilization of oxide-derived third-body debris during sliding. (Nb,Ti)C precipitates act as microscale load-bearing units, contributing to strength enhancement and subsurface damage suppression, but their influence on wear behavior strongly depends on tempering temperature. The dominant wear mechanism is abrasive micro-cutting, accompanied by fatigue-induced spalling and oxidation-assisted damage at later stages. These results demonstrate that wear performance cannot be correlated with hardness alone, but instead requires the coordinated optimization of carbide precipitation and tempered microstructural stability. This work provides microstructural guidance for the design of microalloyed martensitic wear-resistant steels.

## 1. Introduction

Wear is one of the most common and unavoidable failure modes of engineering materials [1,2,3], and it is ubiquitous in service conditions such as mining conveying, construction machinery, metallurgical rolling, and energy equipment. Wear not only shortens component lifetime and increases maintenance costs but also causes substantial energy loss and associated carbon emissions. Under service conditions involving heavy loads, combined impact–sliding, and media containing hard particles, the material surface undergoes a coupled wear process governed by multiple mechanisms, including adhesive wear, abrasive wear, oxidative wear, and fatigue-induced spalling [4]. The associated damage evolution typically follows a progressive failure route of “near-surface plastic deformation → subsurface crack initiation → localized delamination/spalling.” Therefore, developing wear-resistant steels that simultaneously exhibit high load-bearing capacity, impact resistance, and wear resistance [5] is of great significance for improving equipment reliability and reducing life-cycle costs.

At present, the most widely used wear-resistant steel system in engineering practice remains low-alloy martensitic wear-resistant steel. A typical processing route involves obtaining a high-hardness martensitic matrix by quenching, followed by tempering to achieve a compromise between hardness and toughness. However, extensive studies and engineering practice have consistently indicated that although hardness is highly correlated with wear resistance [6], it is not the sole determining factor [7]. Under impact or impact–sliding conditions, sufficient toughness and crack resistance are likewise critical [8,9]. This implies that the design of wear-resistant steels should not be limited to “simply pursuing high hardness” but should instead be systematically optimized with a focus on strength–toughness matching and microstructural stability.

From the viewpoint of microstructural control, tempering is accompanied by dislocation recovery, polygonization, and cementite precipitation and coarsening, which can readily lead to softening of the martensitic matrix [10]. At higher tempering temperatures or with longer tempering times, the softening effect is often more pronounced. Therefore, enhancing resistance to tempering softening and achieving a uniform, crack-resistant tempered martensitic microstructure are key to synergistic strength–toughness–wear improvement.

Microalloying provides an effective pathway to strengthen and toughen wear-resistant steels [11,12,13]. In particular, strong carbide-forming elements such as Ti and Nb can form fine and dispersed MC-type carbides (e.g., TiC, NbC, and (Ti,Nb)C) [14], thereby providing precipitation strengthening and microstructural stabilization during phase transformation and heat treatment. Nanoscale carbides enhance matrix strength via dislocation pinning, while their precipitation characteristics suppress tempering-induced softening and refine the microstructure. Particularly in quenched and tempered systems, if fine and uniformly dispersed (Ti,Nb)C precipitates can be achieved [15,16,17] and act synergistically with the dislocation substructure of tempered martensite and the evolution of cementite, it becomes possible to retain the necessary plasticity, toughness, and crack resistance at relatively high hardness levels, thereby enhancing subsurface resistance to spalling and the overall wear resistance during sliding wear.

Existing studies have accumulated important understanding of the microstructures and wear behaviors of Ti/Nb microalloyed wear-resistant steels. For example, Ti-involved carbide precipitation has been reported to enhance the stability of tempered microstructures and improve wear resistance [18,19], and martensitic wear-resistant steels exhibit characteristic sliding-wear responses under different temperatures or tribological conditions. Nevertheless, the effects of tempering parameters on carbide evolution, dislocation stability, and wear mechanisms remain insufficiently understood [20].

Despite the progress made, several critical gaps remain. First, most existing studies focus on either the effect of tempering on mechanical properties or the wear response under single conditions, without systematically linking the coupled evolution of MC-type carbides and tempered martensite to stage-dependent wear mechanisms. Second, the role of oxide-derived third bodies in dry-sliding wear has been noted but not quantitatively analyzed in relation to the destabilization of (Nb,Ti)C precipitates and the resulting non-monotonic wear kinetics. Third, while hardness is often used as a proxy for wear resistance, the synergistic matching of strength, toughness, and carbide stability—especially in Nb-containing systems—remains poorly understood. To address these gaps, the present study systematically investigates three low-carbon martensitic steels with different Ti/Nb microalloying strategies, subjected to a range of tempering conditions.

To clarify the nature of the contribution, this work proposes a mechanistic framework in which the “coupling mechanism” refers to the synergistic interplay between MC-type carbide characteristics (size, distribution, lattice misfit) and tempered matrix properties (hardness, impact toughness) that governs the stage-dependent wear transition from abrasive micro-cutting to fatigue-induced spalling and finally to oxidation-assisted third-body involvement. Thus, the manuscript does not present merely an empirical alloy-by-alloy comparison, but rather a testable mechanistic model based on carbide-microstructure-property linkages.

Based on this, the present study designs a heat-treatment window centered on ‘carbide precipitation + synergistic control of tempered microstructure. By combining microstructural and precipitate characterization with mechanical property evaluation and friction/wear testing, we systematically assess the intrinsic correlations among strength, hardness, impact toughness, and wear performance [18,21], and elucidate the coupled effects of (Ti,Nb)C precipitation and tempered microstructures on wear mechanisms (e.g., abrasive ploughing, oxide film formation/breakdown, and subsurface crack propagation) [18,22,23]. Wear performance is comparatively evaluated using a pin-on-disk/standard-like sliding wear method, and hardness and impact tests are conducted with reference to relevant international standards. In this work, the ‘coupling mechanism’ is operationally defined as a causal chain that explicitly links material parameters to wear performance: carbide characteristics (type, size, distribution, lattice misfit) → tempered martensite properties (hardness, impact toughness) → stage-dependent wear mechanism (abrasive micro-cutting, fatigue-induced spalling, or oxidation-assisted third-body involvement). This causal framework distinguishes the present work from purely correlative analyses and provides a testable mechanistic basis for understanding strength-toughness-wear coupling in microalloyed martensitic steels.

## 2. Materials and Experimental Methods

### 2.1. Materials Design and Preparation

Three low-alloy wear-resistant steels with different compositions were investigated in this work, namely 0.04Ti, 0.04Ti/Nb, and 0.1Ti (see Table 1 for chemical compositions). The Ti and Nb contents were selected within typical industrial ranges for microalloyed wear-resistant steels (Ti: 0.02–0.12 wt%, Nb: 0.02–0.06 wt%) [24]. The 0.04Ti and 0.1Ti compositions allow comparison of moderate vs. high Ti addition, while the 0.04Ti/Nb composition is designed to evaluate the combined effect of Ti and Nb. The experimental steels were melted and refined in a 50 kg vacuum induction furnace and cast into ingots with dimensions of 60 mm × 65 mm × 150 mm.

The ingots were hot-rolled into plates using a Φ450 mm two-high reversible hot-rolling mill. To ensure comparable microstructures, rolling was carried out using a seven-pass, two-stage controlled rolling schedule: the first stage was performed in the austenite recrystallization region (starting rolling temperature: 1100 °C), and the second stage was performed in the austenite non-recrystallization region (finishing rolling temperature: 840 °C). The thickness reduction schedule was 60 → 42 → 29 → 21 → 15 → 11 → 8 → 6 mm.

After rolling, the cooling rate was controlled by adjusting the water flow of laminar cooling, and the steels were produced using a controlled rolling and controlled cooling process. The plates were cooled to 200–300 °C and then placed in an insulation tank for slow cooling to promote alloy carbide precipitation and stabilize the microstructure.

### 2.2. Heat-Treatment Schedules and Sample Designation

The hot-rolled plates were austenitized at 920 °C, held, and then water-quenched to obtain a martensitic matrix. Subsequent tempering was applied to regulate strength–toughness matching and carbide precipitation behavior. The heating rate for austenitization was approximately 10 °C/min, as controlled by the furnace program. After austenitizing at 920 °C for 15 min, the samples were immediately water-quenched; the quenching medium was room-temperature circulating water, and the transfer time from furnace to water was less than 5 s. Tempering was conducted in a laboratory-scale resistance furnace under ambient air atmosphere. No protective atmosphere was used, as the short duration (30 min) and relatively low temperatures (200–550 °C) result in only superficial oxidation, which does not affect the core microstructure or mechanical properties. These parameters were kept consistent across all samples to ensure comparability. For clarity, quenched specimens are denoted as “Q–composition” (e.g., Q-0.04Ti), and low-temperature tempered specimens are denoted as “T–composition” (e.g., T-0.04Ti).

For baseline comparison, specimens were treated by water quenching at 920 °C for 15 min followed by tempering at 200 °C for 30 min to relieve quenching residual stresses and obtain a tempered martensite microstructure. In addition, the 0.04Ti/Nb steel was tempered at different temperatures (200 °C, 350 °C, and 550 °C) to investigate the chain of “tempered microstructure evolution/carbide precipitation → strength–toughness matching → wear resistance.”

The selected tempering temperatures—200 °C, 350 °C, and 550 °C—represent three distinct regimes of microstructural evolution. Tempering at 200 °C (low-temperature) relieves quenching residual stresses while largely preserving the high dislocation density and fine lath structure of martensite, with only limited carbide precipitation. At 350 °C (intermediate), martensite decomposes into tempered troostite, accompanied by more extensive carbide precipitation and partial recovery of dislocations. At 550 °C (high-temperature), the microstructure transforms into tempered sorbite with coarsened carbides and significant dislocation annihilation, leading to lower strength but higher toughness. This range allows systematic investigation of the transition from strength-dominated to toughness-dominated wear behavior.

### 2.3. Microstructural and Second-Phase Characterization

Metallographic specimens were taken from the mid-thickness position of the plates (approximately 6 mm × 5 mm × 6 mm). After stepwise grinding and polishing, the specimens were etched with 4% nital for microstructural observation (Olympus BX51, Olympus Corporation, Tokyo, Japan). Phase analysis was performed by X-ray diffraction (XRD, Bruker D8 Advance, Bruker AXS, Karlsruhe, Germany); the tested surface was ground and polished prior to measurement. The 2θ range was 40–120°, and the scan rate was 2°/min.

Microstructures, impact fracture surfaces, and worn morphologies were examined by scanning electron microscopy (SEM, ZEISS Gemini360, Carl Zeiss, Oberkochen, Germany), combined with energy-dispersive spectroscopy (EDS) for compositional analysis. The morphology and structure of second phases were further characterized by transmission electron microscopy (TEM, FEI Talos F200X, Thermo Fisher Scientific, Hillsboro, OR, USA). TEM foils were prepared by mechanical thinning followed by disc punching, twin-jet electropolishing (Struers TenuPol-5, Struers, Ballerup, Denmark), and ion-beam polishing (Gatan Model 695 PIPS, Gatan, Pleasanton, CA, USA) to obtain electron-transparent regions.

### 2.4. Mechanical Property Testing

Tensile tests were performed on a CMT5105 universal testing machine (Shenzhen SANS Testing Machine Co., Ltd., Shenzhen, China) at a crosshead speed of 2 mm·min^−1^ using specimens machined along the rolling direction. An extensometer was used to record deformation. At least three parallel specimens were tested per condition, and the average value was reported. Charpy V-notch impact toughness was tested according to the national standard [25] using a JBN-300B pendulum impact tester (Jinan Shijin Group, Jinan, China); at least three repeats were performed, and the average absorbed energy was taken. Hardness was measured using a Brinell tester (Shanghai Hualong Testing Instruments Co., Ltd., Shanghai, China) (1500 N, 15 s) on 20 mm × 20 mm ground specimens, with five indents per condition; the average value was calculated.

Statistical significance was not calculated because the sample sizes are limited. The reported mean ± SD values are descriptive, and comparisons between close values are made with caution.

### 2.5. Pin-on-Disk Dry-Sliding Wear Test and Data Processing

**Figure 1:**
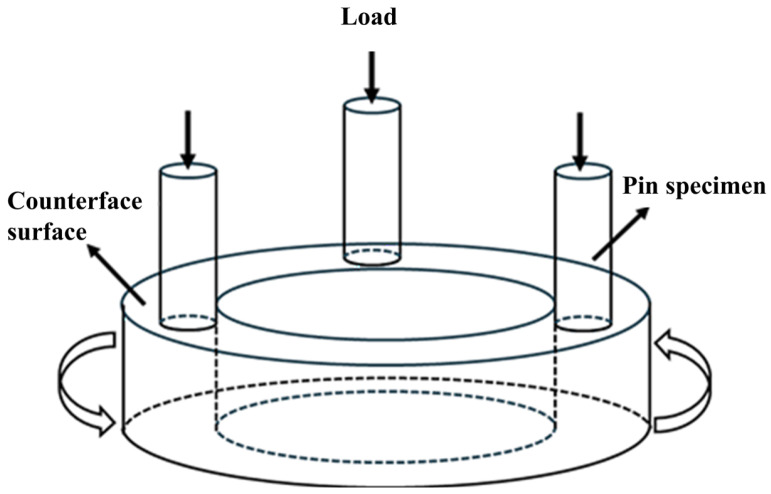
Schematic diagram of the pin-on-disk wear testing machine.

Wear tests were conducted on an MMD-1 multifunctional friction and wear testing machine (Jinan Yihua Tribology Testing Technology Co., Ltd., Jinan, China) under a dry sliding configuration (Figure 1). The specimens were machined into friction pins measuring Φ4.8 mm × 15 mm. The counter disk was made of quenched 45 steel (hardness approximately 420 HB, diameter 44 mm). Prior to testing, both pin and disk surfaces were ground to 1000 grit. The friction pins were ultrasonically cleaned and pre-worn for 5 min before their initial mass was measured using an electronic balance (Mettler Toledo, Columbus, OH, USA) with an accuracy of 0.0001 g. Tests were performed under normal loads of 50 N and 100 N, with a spindle speed of 100 rpm, resulting in a linear speed of approximately 0.19 m·s^−1^. These normal loads were selected based on preliminary tests to ensure that the wear process occurs primarily in the mild-to-moderate wear regime without causing gross plastic deformation or seizure of the pin sample. The lower load (50 N) represents relatively mild sliding conditions typical of lightly loaded components, while the higher load (100 N) represents more severe service conditions encountered in mining and construction equipment. These values are within the typical range used for pin-on-disk testing of martensitic wear-resistant steels. All wear tests were conducted under ambient laboratory conditions (room temperature, uncontrolled humidity). Because the tested steels are not highly sensitive to moderate variations in temperature and humidity under dry sliding conditions, the influence of these environmental factors on the comparative results is considered minor. Only one counterface material (quenched 45 steel) was used; therefore, caution is needed when generalizing the results to other wear conditions. Sliding was conducted under dry conditions for 60 min (total sliding distance of approximately 680 m). Wear mass was measured at intervals of 15, 30, 45, and 60 min by stopping the test. The wear mass loss is defined as

(1) $$\Delta m(t)=m_{0}-m_{t}$$

where $m_{0}$ and $m_{t}$ are the masses before wear testing and at time t, respectively. Each condition was tested at least three times, and the results are presented as “mean ± standard deviation”.

To facilitate the comparison of wear responses among different specimens under identical testing conditions, the average wear rate is defined as

(2) $$v_{m}=\frac{∆m(t)}{t}$$

where t is measured in minutes. Thus, $v_{m}$ has units of mg/min. Furthermore, the relative wear resistance index is introduced:

(3) $$I_{w}=\Delta m_{ref}/\Delta m_{i}$$

where $\Delta m_{\mathrm{ref}}$ is the wear mass loss of the reference specimen under identical testing conditions. where $\Delta m_{i}$ is the wear mass loss of the i-th test specimen. A higher value of $I_{w}$ indicates better wear resistance.

### 2.6. Variable Design and Control

Composition comparison (0.04Ti vs. 0.04Ti/Nb vs. 0.1Ti): the controlled rolling and controlled cooling schedules were kept identical (starting/finishing rolling temperatures, pass reductions, final cooling temperature range, and cooling rate system), and the heat treatment was fixed as “quenching + low-temperature tempering.” The performance differences are mainly attributed to differences in Ti/Nb carbide precipitation behavior and tempered microstructure regulation.

Tempering temperature comparison (0.04Ti/Nb: 200/350/550 °C): the chemical composition and controlled rolling/controlled cooling conditions were kept identical, and only the tempering temperature was varied, thereby strengthening the logical expression of “tempered microstructure evolution → strength–toughness matching → wear response.”

## 3. Results and Discussion

### 3.1. Microstructure and Mechanical Properties of the Experimental Steels

**Figure 2:**
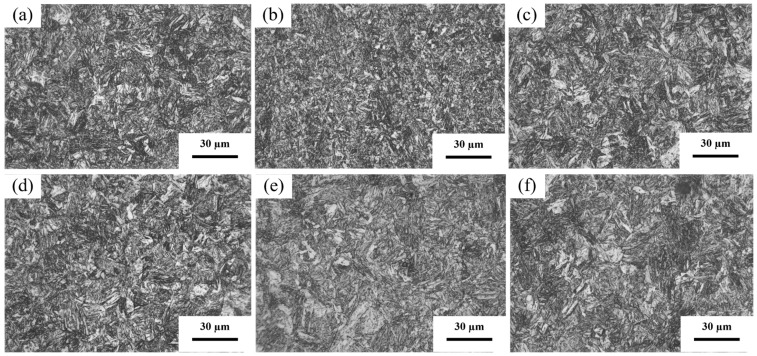
Microstructures of the experimental steels in the quenched and tempered conditions. (**a**) Q-0.04Ti; (**b**) Q-0.1Ti; (**c**) Q-0.04Ti/Nb; (**d**) T-0.04Ti; (**e**) T-0.1Ti; (**f**) T-0.04Ti/Nb.

Figure 2 shows the SEM microstructures of the experimental steels in the quenched and tempered conditions. As shown in Figure 2a–c, all three experimental steels are composed of quenched martensite. With increasing alloying content, the microstructure becomes noticeably refined. This refinement is mainly attributed to the strong affinity of Ti and Nb for carbon, leading to the formation of second-phase TiC and (Ti,Nb)C carbides [26]. These carbide particles are known to create local dislocation structures around them, which can contribute to strengthening. This is inferred from the observed hardness increase and is consistent with literature reports on Ti/Nb-containing steels [27,28]. As a result, the number of martensite nucleation sites during quenching increases, leading to microstructural refinement and a significant enhancement in hardness and strength.

Figure 2d–f present the SEM microstructures of the three experimental steels after tempering. All the tempered steels consist of tempered martensite. Compared with the quenched condition, the microstructure in the tempered state becomes slightly coarser, indicating that partial martensite decomposition occurs during tempering. Meanwhile, increased carbide precipitation strengthens the microstructure while simultaneously improving plasticity and toughness.

**Figure 3:**
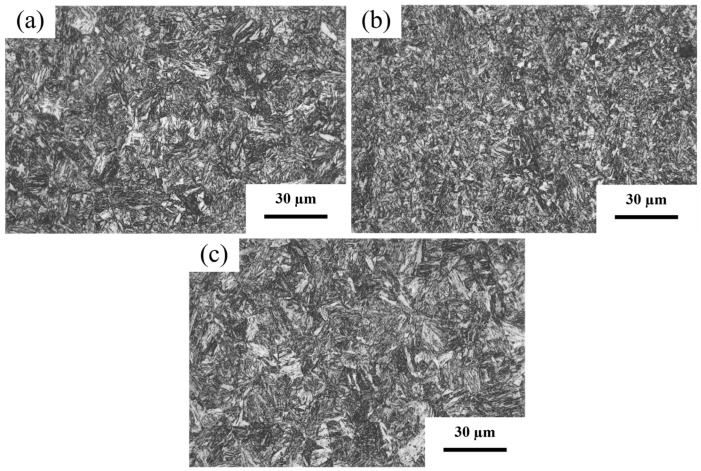
Microstructures of the 0.04Ti/Nb experimental steel tempered at different temperatures. (**a**) 200 °C; (**b**) 350 °C; (**c**) 550 °C.

Figure 3 shows the SEM microstructures of the 0.04Ti/Nb experimental steel tempered at different temperatures. With increasing tempering temperature, the martensitic structure gradually decomposes and transforms into tempered troostite and tempered sorbite, accompanied by progressive microstructural refinement and increased carbide precipitation. This indicates that the tempering temperature has a pronounced influence on both the microstructure and the carbide precipitation behavior of the experimental steel [29,30]. The significant effect of tempering temperature on microstructural refinement and carbide distribution directly contributes to improved toughness and enhanced wear resistance of the tempered experimental steel.

**Figure 4:**
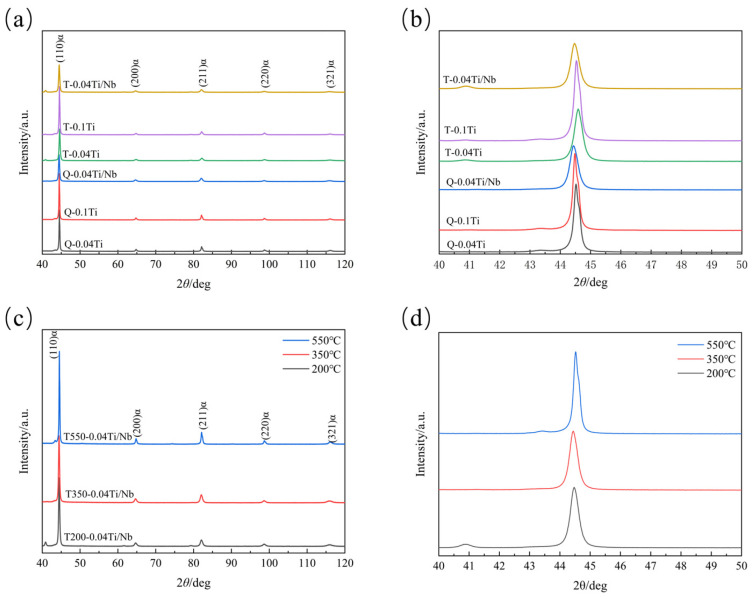
XRD patterns of the experimental steels. (**a**) XRD patterns of the experimental steels with different compositions after quenching and tempering; (**b**) enlarged views of the XRD patterns of the experimental steels with different compositions; (**c**) XRD patterns of the 0.04Ti/Nb experimental steel tempered at different temperatures; (**d**) enlarged views of the XRD patterns of the 0.04Ti/Nb experimental steel tempered at different temperatures.

Figure 4 presents the XRD patterns of the experimental steels. The diffraction peaks of all specimens are dominated by α-Fe, and no distinct γ-Fe characteristic peaks are observed within the present testing conditions and detection limits, indicating a relatively low content of retained austenite. It should be noted that the finely and discretely precipitated phases, such as TiC/(Nb,Ti)C, possess very small particle sizes and limited volume fractions, making it difficult for XRD to provide clear carbide diffraction peaks. Therefore, phase identification in this study is discussed in conjunction with subsequent selected-area electron diffraction (SAED) and lattice-spacing analyses obtained from HRTEM observations.

A slight shift in the α-Fe main peaks is observed for different compositions and tempering conditions (Figure 4b,d). The shifts could be related to lattice parameter changes induced by Ti/Nb solid solution, carbon precipitation, or residual stress variations. As the tempering temperature increases, the peaks tend to shift back to higher angles, which might indicate a decrease in interplanar spacing, possibly associated with carbon partitioning and stress relaxation. Because XRD peak positions are influenced by multiple factors, this discussion remains qualitative and is consistent with the subsequent results on carbide precipitation and mechanical property evolution.

**Figure 5:**
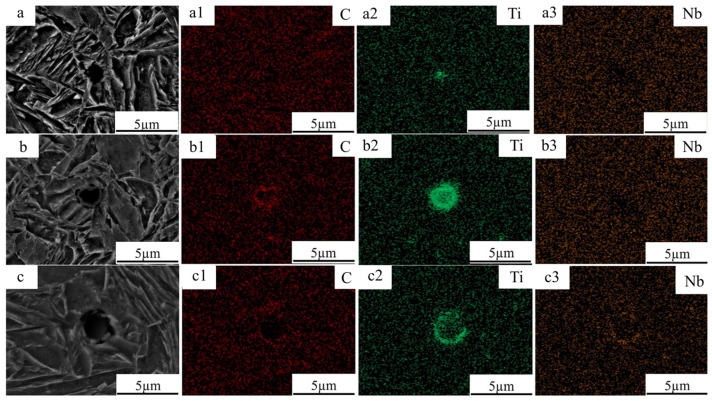
EDS spectra of the experimental steels with different compositions. (**a**) T-0.04Ti; (**b**) T-0.1Ti; (**c**) T-0.04Ti/Nb. (**a1**) Carbon (C) spectrum of T-0.04Ti (red); (**a2**) titanium (Ti) spectrum of T-0.04Ti (green); (**a3**) niobium (Nb) spectrum of T-0.04Ti (orange); (**b1**) C spectrum of T-0.1Ti (red); (**b2**) Ti spectrum of T-0.1Ti (green); (**b3**) Nb spectrum of T-0.1Ti (orange); (**c1**) C spectrum of T-0.04Ti/Nb (red); (**c2**) Ti spectrum of T-0.04Ti/Nb (green); (**c3**) Nb spectrum of T-0.04Ti/Nb (orange).

Because carbide precipitation occurs in the experimental steels, EDS analyses were conducted on the three steels to determine the composition of the precipitates (as shown in Figure 5). According to the EDS results, the carbide particles in the 0.04Ti and 0.1Ti experimental steels are mainly enriched in Ti and C, whereas the carbide particles present in the matrix of the 0.04Ti/Nb experimental steel are primarily enriched in C, Ti, and Nb. Based on these results, the precipitated carbides can be preliminarily identified as TiC in the 0.04Ti and 0.1Ti steels, and as (Nb,Ti)C in the 0.04Ti/Nb steel.

**Figure 6:**
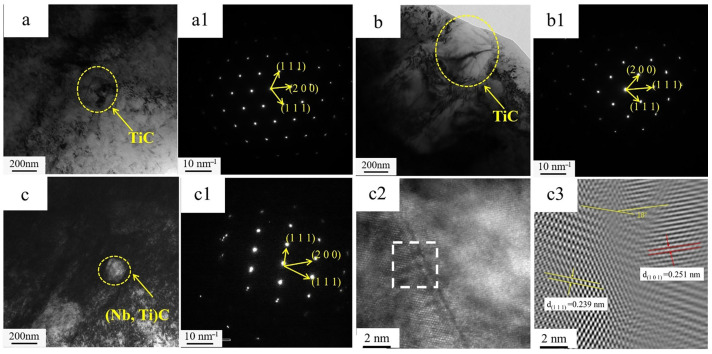
TEM images of the experimental steels with different compositions. (**a**) T-0.04Ti; (**b**) T-0.1Ti; (**c**) T-0.04Ti/Nb. (**a**–**c**) Morphologies of second-phase particles; (**a1**,**b1**,**c1**) selected-area electron diffraction (SAED) patterns of the corresponding particles; (**c2**) high-resolution TEM image of the (Nb,Ti)C/matrix interface; (**c3**) inverse fast Fourier transform (IFFT) image of the region marked by the white box in (**c2**).

It should be noted that EDS analysis is mainly used to identify elemental enrichment rather than crystal structure. Therefore, the final phase identification of the precipitates in this study is based on selected-area electron diffraction (SAED) and lattice-spacing analyses obtained from HRTEM observations (Figure 6), by which the precipitates are conclusively identified as TiC and (Nb,Ti)C. The precipitation of these carbides plays a critical role in enhancing the mechanical properties of the experimental steels.

To further analyze the phase constitution of the precipitates, transmission electron microscopy (TEM) observations were performed. Figure 6 shows the TEM images of the three experimental steels.

Figure 6a–c show the morphologies of carbide particles in the three steels, and Figure 6(a1,b1,c1) present their electron diffraction patterns. Combined with PDF standard cards, the particles are identified as TiC in the 0.04Ti and 0.1Ti steels and as (Nb,Ti)C in the 0.04Ti/Nb steel. The average diameters measured from TEM images using ImageJ (version 1.54p, National Institutes of Health, Bethesda, MD, USA) are about 200 nm (0.04Ti), 400 nm (0.1Ti), and 100 nm (0.04Ti/Nb), respectively, indicating that Nb suppresses particle coarsening. Figure 6(c2) shows a high-resolution TEM image of the (Nb,Ti)C/matrix interface, and Figure 6(c3) is the IFFT image of the white-boxed region. The FFT pattern reveals a stable orientation relationship between α-Fe and (Nb,Ti)C, with an angle of about 18° between the corresponding diffraction vectors. Martensite lath widths were measured from high-resolution SEM images using ImageJ. The average widths are approximately 550 nm (Q-0.04Ti), 430 nm (Q-0.1Ti), and 510 nm (Q-0.04Ti/Nb). Other quantitative parameters were not measured due to the exploratory scope and limited statistically representative images.

This angle is used only to characterize the relative orientation relationship of the two phases in the local region and serves as qualitative evidence for the presence of an orientation relationship at the interface; it is not involved in the quantitative evaluation of lattice misfit. The quantitative assessment of interfacial misfit is instead based on the difference in the corresponding interplanar spacings. The interplanar spacings of the (101) plane of the α-Fe matrix and the (111) plane of (Nb,Ti)C are d_α_ = 0.251 nm and d _(Nb,Ti)C_ = 0.239 nm, respectively. To quantitatively characterize the geometric matching degree of the interface between the two phases, the interplanar spacing misfit parameter ω is defined as follows:

(4) $$\omega =\left|\frac{d_{a}-d_{\left(Nb,Ti\right)C}}{d_{\left(Nb,Ti\right)C}}\right|$$

Substituting the interplanar spacings ($d_{\alpha }=0.251$ nm, $d_{(\mathrm{Nb},\;\mathrm{Ti})\;C}=0.239$ nm) gives ω≈0.050.

The ω value falls between 0.05 and 0.25, indicating the formation of a semi-coherent interface between the BCC phase and the (Nb,Ti)C particles [31], and suggesting good interfacial bonding between the precipitates and the matrix. Therefore, the presence of a semi-coherent interface, together with the increased dislocation density, is expected to play an important role in improving the mechanical properties of the material.

**Figure 7:**
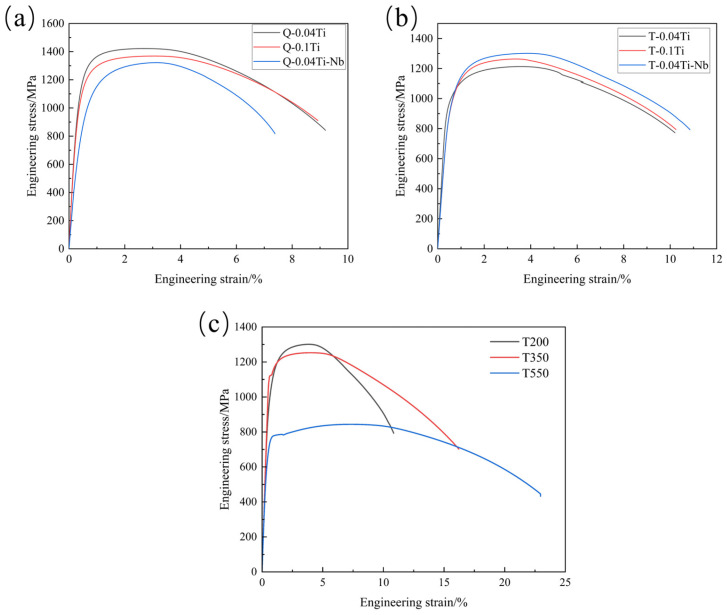
Engineering stress–strain curves of the experimental steels. (**a**) Quenched steels with different compositions; (**b**) tempered steels with different compositions; (**c**) 0.04Ti/Nb steel tempered at different temperatures.

Figure 7 shows the engineering stress–strain curves of the experimental steels, and Table 2 summarizes the mechanical properties of the experimental steels in the quenched and tempered conditions. The alloying elements Ti and Nb enhance the strength of the experimental steels through two primary mechanisms: on the one hand, they combine with carbon to form carbides, thereby increasing dislocation density and strengthening the matrix; on the other hand, they dissolve in the matrix, leading to enhanced lattice distortion and consequently higher strength. Compared with the quenched condition, the tempered steels exhibit reduced strength and hardness but improved toughness. This behavior is attributed to the reduction in quenching-induced residual stresses and the enhancement of plasticity and toughness after low-temperature tempering [32,33].

It should be noted that the yield strength of the T-0.04Ti/Nb steel is higher than that of its quenched counterpart (Table 2). This combination of increased yield strength but decreased tensile strength/hardness can be understood as the result of competition between precipitation strengthening and matrix softening during low-temperature tempering. For the 0.04Ti/Nb system, the strong affinity of Nb/Ti for carbon promotes the preferential precipitation of fine (Nb,Ti)C/TiC particles at dislocations, lath boundaries, and subgrain boundaries, forming a high number density of finely dispersed second-phase particles. These precipitates pin dislocation motion, providing a stronger strengthening contribution at yielding and leading to an apparent increase in yield strength. Meanwhile, tempering reduces residual stresses and induces recovery of the dislocation structure, which weakens work-hardening capability and decreases tensile strength and hardness. Thus, during yielding, the increment from precipitation strengthening may exceed the decrement from matrix softening, while at higher strains the softening effect dominates. However, we acknowledge that direct evidence of increased precipitate density or a quantified strengthening contribution is not available; the proposed competition remains a plausible interpretation but should be considered speculative. To avoid misinterpretation, the relationship between strength, hardness, toughness, and wear resistance is discussed in terms of their combined matching effect, rather than treating any single parameter as a direct determinant of wear resistance.

Table 3 lists the mechanical properties of the 0.04Ti/Nb experimental steel tempered at different temperatures. With increasing tempering temperature, the yield strength and ultimate tensile strength of the experimental steel decrease markedly, whereas plasticity and toughness are significantly improved. In addition, as can be readily observed from Figure 7b, the engineering stress–strain curves of the experimental steel gradually exhibit a yield plateau with increasing tempering temperature, and the extent of the yield region becomes progressively larger. This behavior is mainly attributed to the fact that, at low tempering temperatures, the tempered martensitic microstructure contains a high density of dislocations and twins, accompanied by severe lattice distortion, which hinders dislocation motion [34]. Under such conditions, the yielding process is readily masked by strengthening mechanisms such as grain refinement and solid-solution strengthening. As the tempering temperature increases, martensite progressively decomposes and the dislocation density in the experimental steel decreases, facilitating the formation of pinned dislocations. Once unpinned, these dislocations can continue to glide under an approximately constant applied stress, resulting in pronounced plastic deformation until their motion is impeded by new obstacles (such as grain boundaries or work hardening), thereby giving rise to a yield plateau.

Overall, the relationship between microstructure and mechanical properties of the experimental steels is highly complex. The addition of Ti and Nb not only refines the microstructure through carbide precipitation but also enhances strength via solid-solution strengthening and increased lattice distortion. During tempering, carbide precipitation and martensitic transformation play key roles in improving toughness and wear resistance. The wear resistance of the experimental steels is enhanced after tempering treatment, particularly at low tempering temperatures, where the balance between strength/hardness and toughness becomes a decisive factor.

### 3.2. Analysis of Impact Fracture Morphology

#### 3.2.1. Comparison of Impact Fracture Morphologies in the Quenched and Tempered Conditions

**Figure 8:**
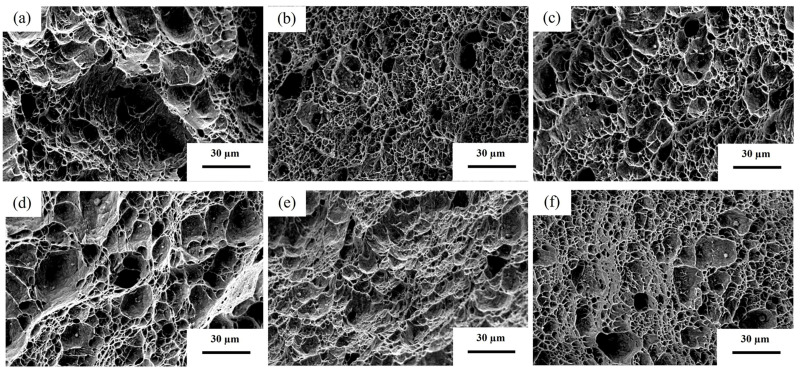
Charpy impact fracture morphologies of the experimental steels in the quenched and tempered conditions. (**a**) Q-0.04Ti; (**b**) Q-0.1Ti; (**c**) Q-0.04Ti/Nb; (**d**) T-0.04Ti; (**e**) T-0.1Ti; (**f**) T-0.04Ti/Nb.

Figure 8 presents the Charpy impact fracture morphologies of the experimental steels in the quenched (Q) and tempered (T) conditions. The fracture surfaces of the quenched experimental steels (Figure 8a–c) all exhibit typical dimpled morphologies; however, significant differences are observed in dimple size and distribution.

For Q-0.04Ti (Figure 8a), the dimples are relatively large and uniformly distributed, with an average diameter of about 9.8 μm and considerable depth, indicating strong plastic deformation capacity and high impact toughness (124 J·cm^−2^). The moderate Ti content forms fine TiC particles that strengthen the matrix without severely damaging its continuity, allowing active dislocation motion.

In contrast, the dimples in Q-0.1Ti (Figure 8b) and Q-0.04Ti/Nb (Figure 8c) are significantly smaller (average diameters ~5.6 μm and ~7.1 μm, respectively) and more sparsely distributed. Q-0.04Ti/Nb also exhibits local quasi-cleavage features. These observations correspond to their lower impact toughness values (108 J·cm^−2^ and 112 J·cm^−2^). The differences are mainly attributed to the increased precipitation of hard carbides (TiC and (Nb,Ti)C). In Q-0.1Ti, excessive Ti leads to coarser TiC particles, which act as crack initiation sites and impede long-range dislocation motion, increasing brittleness. In Q-0.04Ti/Nb, the simultaneous addition of Ti and Nb forms more complex (Nb,Ti)C carbides. Although these carbides refine the microstructure, the higher volume fraction of hard phases restricts plastic deformation, induces stress concentration, and reduces the material’s plastic deformation capability.

After tempering (Figure 8d–f), the fracture morphologies change markedly. T-0.04Ti (Figure 8d) and T-0.1Ti (Figure 8e) show reduced numbers of dimples but a higher proportion of large dimples (average diameters ~8.7 μm and ~6.9 μm, respectively), accompanied by pronounced tearing ridges. These features indicate enhanced toughness after tempering, with impact toughness increasing to 133 J·cm^−2^ and 128 J·cm^−2^.

For T-0.04Ti/Nb (Figure 8f), the average dimple diameter decreases to about 5.0 μm, but the cleavage facets disappear and the dimple structure becomes more uniform, leading to a modest toughness improvement to 115 J·cm^−2^. This observation is consistent with the behavior of low-temperature tempered martensite, where fine carbide precipitation can refine dimples while eliminating brittle cleavage. The addition of Nb enhances strength through semi-coherent carbide/matrix interfaces, but also constrains large-scale plastic flow, limiting toughness recovery.

#### 3.2.2. Fracture Evolution of the 0.04Ti/Nb Steel at Different Tempering Temperatures

**Figure 9:**
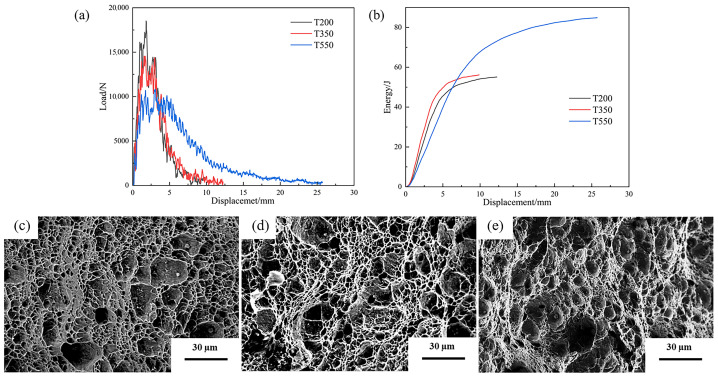
Impact fracture morphologies of the 0.04Ti/Nb experimental steel tempered at different temperatures, together with the impact force–displacement and impact energy–displacement curves after tempering at different temperatures. (**a**) Impact force–displacement curves of the experimental steel; (**b**) impact energy–displacement curves of the experimental steel; (**c**) T200; (**d**) T350; (**e**) T550.

Figure 9 further presents the fracture morphologies of the 0.04Ti/Nb experimental steel tempered at different temperatures. The fracture surface of the steel tempered at 200 °C (T200-0.04Ti/Nb, Figure 9a) is dominated by shallow dimples accompanied by local cleavage facets [35,36], which is similar to that observed in the quenched condition, indicating that low-temperature tempering provides only limited improvement in microstructural plasticity. For the steel tempered at 350 °C (T350-0.04Ti/Nb, Figure 9b), both the size and depth of dimples increase markedly, and their distribution becomes more continuous, corresponding to an increase in impact toughness to 116 J·cm^−2^. In contrast, the fracture surface of the steel tempered at 550 °C (T550-0.04Ti/Nb, Figure 9c) exhibits typical ductile fracture characteristics, with further enlarged dimples (approximately 15–20 μm in diameter), uniform dimple depth, and no observable cleavage features, which is consistent with its highest impact toughness value of 178 J·cm^−2^. This evolution results from high-temperature tempering, which promotes the decomposition of martensite into tempered sorbite and the dispersed precipitation of carbides, thereby significantly enhancing the plastic deformation capability of the material.

### 3.3. Wear Resistance

#### 3.3.1. Wear Mass Loss

**Figure 10:**
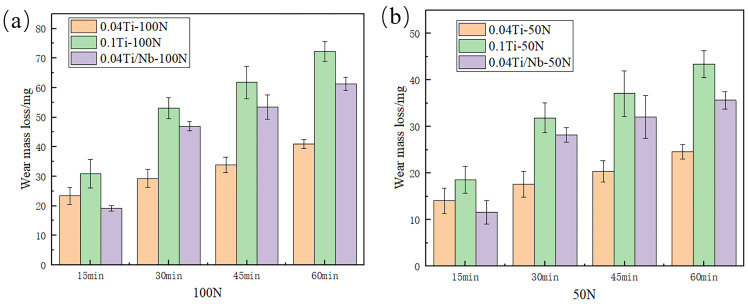
Wear mass loss of the tempered experimental steels with different compositions under different applied loads. (**a**) Under a load of 100 N; (**b**) under a load of 50 N.

Figure 10 shows the time-dependent wear mass loss of the three experimental steels with different compositions under loads of 50 N and 100 N. To avoid empirical judgments based solely on terminal values, the wear process is quantitatively compared by combining the final wear loss with segmented incremental mass losses, as summarized in Table 4.

Under a load of 100 N for 60 min, the wear mass losses of the quenched specimens are 63.8 ± 2.2 mg for Q-0.04Ti, 128.3 ± 4.5 mg for Q-0.1Ti, and 52.2 ± 5.1 mg for Q-0.04Ti/Nb (Table 4). After low-temperature tempering, the wear mass losses of T-0.04Ti and T-0.1Ti decrease to 41.0 ± 1.5 mg and 72.3 ± 3.4 mg, corresponding to reductions of 35.7% and 43.6%, respectively, compared with their quenched counterparts (Table 4). These results indicate that, for the 0.04Ti and 0.1Ti compositions, the tempered martensitic microstructure obtained by low-temperature tempering, together with precipitation strengthening, can effectively reduce dry-sliding wear loss. In contrast, the wear mass loss of T-0.04Ti/Nb reaches 71.1 ± 4.2 mg, which is higher than that of Q-0.04Ti/Nb (52.2 ± 5.1 mg) (Table 4). This suggests that the wear response of the Nb-containing system is not linearly governed by a single strength or hardness parameter, but instead requires interpretation in conjunction with the formation and stability of third bodies/oxide products and their spalling behavior during wear (see Section 3.3.2).

From the perspective of time evolution, T-0.04Ti/Nb exhibits pronounced stage-dependent behavior. Its wear mass loss during the first 0–30 min already accounts for approximately 66% of the total loss at 60 min. The segmented incremental mass losses are 47.0 ± 1.65 mg for 0–30 min, +6.5 mg for 30–45 min (from 47.0 to 53.5 ± 4.1 mg), and +17.6 mg for 45–60 min (from 53.5 to 71.1 ± 4.2 mg) (Table 4). This evolution, characterized by “mid-stage deceleration followed by late-stage re-acceleration,” is corroborated by subsequent wear scar morphologies and EDS evidence of oxygen enrichment (see Section 3.3.2). It indicates that, during the intermediate stage of wear, oxide-derived third bodies or localized oxide films may provide a temporary load-bearing or isolating effect, whereas their rupture and spalling under sustained shear lead to intensified wear at later stages [37,38,39]. Accordingly, a more appropriate mechanistic description for this specimen is a composite wear process dominated by abrasive micro-cutting, with the participation of oxide products that induce stage-dependent wear mitigation followed by failure, rather than a simplified interpretation based on a single “oxidative lubrication” mechanism.

A transverse comparison among the tempered specimens shows that T-0.04Ti exhibits the lowest wear loss and a more gradual increase under the present conditions (60 min: 41.0 ± 1.5 mg, Table 4; Figure 10), indicating a more stable wear response. Although the wear loss of T-0.1Ti is significantly reduced compared with its quenched state, it remains higher than that of T-0.04Ti (60 min: 72.3 ± 3.4 mg, Table 4), suggesting a greater susceptibility to near-surface cumulative damage and local instability under identical tribological conditions (see Section 3.3.2 for supporting evidence).

To improve the comparability of wear results across different studies, the mass loss Δm was converted into volumetric wear loss ΔV, and the specific wear rate k was calculated. The specific wear rate is defined as the volumetric wear per unit normal load and per unit sliding distance:

(5) $$k=\frac{∆V}{F\cdot L}$$

where F is the normal load (N) and L is the sliding distance (m). Since the quantity directly measured in this study is the mass loss Δm, the volumetric wear loss is calculated using the material density ρ:

(6) $$∆V=\frac{∆m}{\rho }$$

By combining the two equations, the following expression is obtained:

(7) $$k=\frac{∆m}{\rho \cdot F\cdot L}$$

where the density ρ is taken as the commonly used approximate value for low-alloy steels, $\rho =7.85\;{g\cdot \mathrm{cm}}^{-3}$, and the sliding distance is approximately 680 m. For the calculations presented in this study, the normal load F=100 N, the sliding distance L≈680 m, and the density $\rho =7.85\;g\cdot {\mathrm{cm}}^{-3}$. We acknowledge that the density of the three steels may vary slightly due to differences in Ti and Nb content; however, the effect on the calculated specific wear rate is negligible (within ±0.5%) and does not affect the comparative conclusions. Based on the calculations, under the condition of 100 N for 60 min, the specific wear rates k of Q-0.04Ti, Q-0.1Ti, and Q-0.04Ti/Nb are approximately $1.20\times 10^{-4}$, $2.40\times 10^{-4}$, and $9.78\times 10^{-5}$ mm^3^·(N·m)^−1^, respectively.

After low-temperature tempering, the k values of T-0.04Ti and T-0.1Ti decrease to approximately 7.70 × 10^−5^ and 1.35 × 10^−4^ mm^3^·(N·m)^−1^, respectively. These converted results are consistent with the conclusions drawn from the mass loss data, further indicating that low-temperature tempering provides a stable wear reduction benefit for the 0.04Ti and 0.1Ti systems, whereas the wear advantage of the Nb-containing system depends more strongly on its stage-dependent wear behavior and subsurface spalling characteristics.

**Figure 11:**
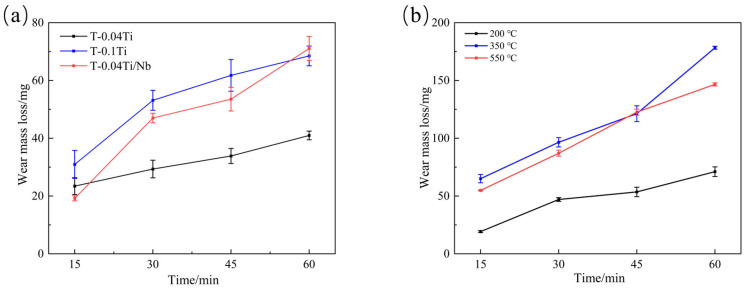
Wear mass loss curves of the experimental steels. (**a**) Tempered experimental steels with different compositions; (**b**) 0.04Ti/Nb experimental steel tempered at different temperatures.

In addition, Figure 11 shows the wear mass loss curves of the tempered experimental steels with three different compositions (Figure 11a) and of the 0.04Ti/Nb experimental steel tempered at different temperatures (Figure 11b), during sliding wear for 60 min under a load of 100 N. As shown in Figure 10 and Table 4, the wear mass loss of the tempered experimental steels is generally lower than that of the quenched steels, indicating that tempering treatment improves the wear resistance of the experimental steels. As can be seen from Table 2, Table 3 and Table 4, the wear resistance generally shows a certain correlation with hardness, indicating that hardness is an important factor. However, as shown by the non-monotonic tempering response of the Nb-containing steel (Figure 11b) and the comparison between T-0.04Ti and T-0.04Ti/Nb, hardness alone does not determine wear resistance; the synergy of strength, toughness, and carbide stability also plays a critical role.

Moreover, the 0.04Ti/Nb system exhibits a non-monotonic response at different tempering temperatures (Figure 11b): under a load of 100 N for 60 min, the wear mass loss of T200-0.04Ti/Nb is 71.1 ± 4.2 mg, which increases to 167.8 ± 1.2 mg for T350, and then decreases to 146.6 ± 1.4 mg for T550 (see Figure 11b). In conjunction with the evolution of mechanical properties shown in Table 3, it can be seen that with increasing tempering temperature, strength and hardness decrease, whereas toughness improves. The T550 condition may partially mitigate wear loss by reducing crack propagation and delamination tendencies, indicating that wear resistance is governed by the combined matching of strength, hardness, and toughness rather than by a single parameter alone (Table 3; Figure 11b) [40,41].

#### 3.3.2. Wear Morphology

To elucidate the differences in wear behavior among the experimental steels with different compositions and heat treatment conditions, comparative analyses were carried out on the worn surface morphologies, subsurface deformation layers in the wear track cross sections, and wear debris/products (EDS). In general, continuous and regular grooves correspond to wear dominated by abrasive micro-cutting/ploughing; layered or flake-like spalling and crack networks typically indicate subsurface crack initiation and propagation induced by contact fatigue; whereas oxygen enrichment or accumulation of oxide products in the wear track suggests the participation of oxidation during wear and the formation of third-body products, whose stability can significantly affect the wear rate and mass loss (see Section 3.3.1).

**Figure 12:**
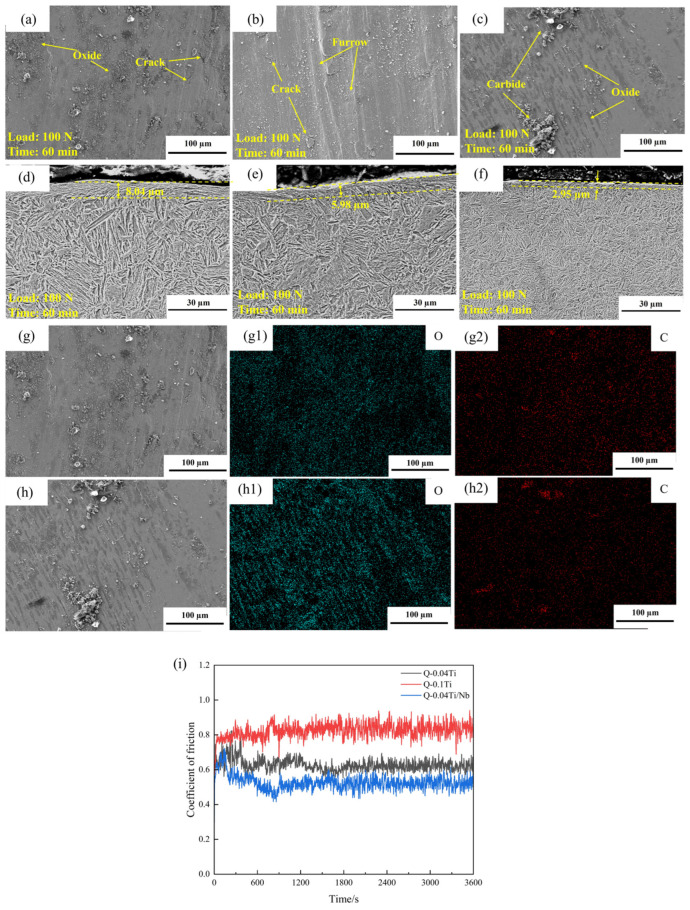
Wear behavior of quenched experimental steels under a load of 100 N(**a**–**f**): worn surface and cross-sectional morphologies of Q-0.04Ti (**a**,**d**), Q-0.1Ti (**b**,**e**), and Q-0.04Ti/Nb (**c**,**f**); (**g**,**h**) EDS spectra of worn surfaces of Q-0.04Ti (**g**) and Q-0.04Ti/Nb (**h**); (**g1**) EDS spectrum of O on the worn surface of Q-0.04Ti; (**g2**) EDS spectrum of C; (**h1**) EDS spectrum of O on the worn surface of Q-0.04Ti/Nb; (**h2**) EDS spectrum of C. (**i**) friction coefficient curves during sliding wear for 60 min.

(1) Quenched specimens (Figure 12)

Figure 12 shows the wear behavior of the quenched specimens, including friction coefficient curves (i), worn surface morphologies (a–c), cross-sectional morphologies (d–f), and EDS spectra (g,h). The wear tracks of all three quenched specimens are characterized by grooves as the basic feature, while notable differences exist in local damage morphologies. The friction coefficients of all three quenched steels fluctuate within the range of 0.4–0.8 and exhibit an overall trend of initial increase followed by stabilization, indicating that the wear process gradually approaches a steady state.

For Q-0.1Ti, in addition to dense grooves, the wear track displays pronounced local tearing and flake-like spalling features. This is consistent with its highest wear mass loss (Table 4) and can be attributed to the presence of coarse TiC particles (from excessive Ti addition), which promote crack initiation and accelerate material removal by fatigue-assisted spalling, and the subsurface deformation zone in the cross section is more continuous (Figure 12e), which is consistent with its highest wear mass loss under 100 N (Table 4). In contrast, the worn surface of Q-0.04Ti/Nb shows more evident coverage/accumulation of oxide products (Figure 12c), and its subsurface deformation layer is thinner (Figure 12f). Meanwhile, EDS mapping reveals pronounced oxygen enrichment in the wear-track region (Figure 12h). Combined with the smaller incremental wear loss of this specimen during the middle and later stages discussed in Section 3.3.1, it can be inferred that this system is more prone to forming oxide-derived third bodies or localized oxide films with a certain load-bearing and isolating effect during wear, thereby reducing direct metal-to-metal contact and suppressing further wear.

**Figure 13:**
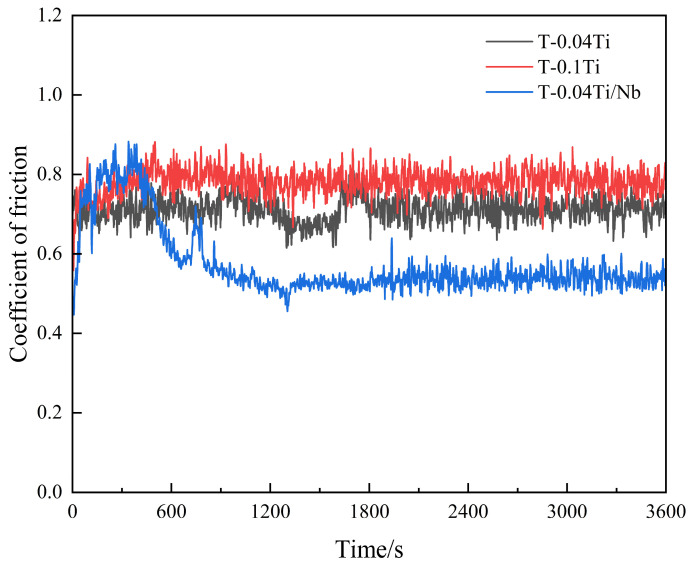
Friction coefficients of the tempered experimental steels.

**Figure 14:**
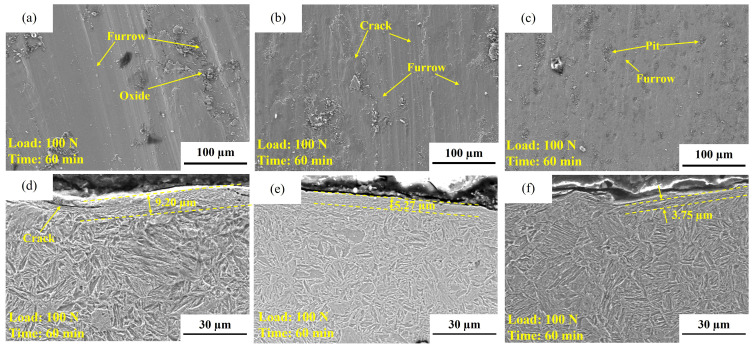
Worn surface and longitudinal cross-sectional morphologies of the tempered experimental steels. (**a**) Worn surface of T-0.04Ti; (**b**) worn surface of T-0.1Ti; (**c**) worn surface of T-0.04Ti/Nb; (**d**) longitudinal cross-section of T-0.04Ti; (**e**) longitudinal cross-section of T-0.1Ti; (**f**) longitudinal cross-section of T-0.04Ti/Nb.

**Figure 15:**
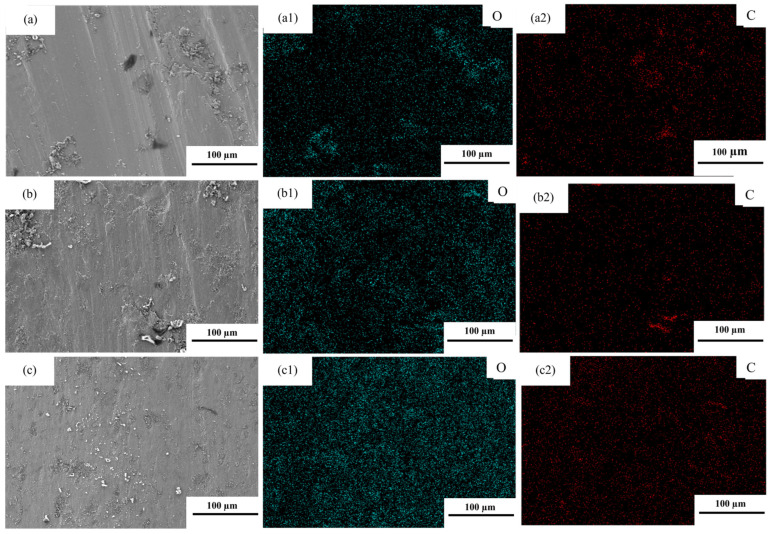
EDS spectra of the tempered experimental steels. (**a**) T-0.04Ti; (**b**) T-0.1Ti; (**c**) T-0.04Ti/Nb. (**a1**) O spectrum of T-0.04Ti (green); (**a2**) C spectrum of T-0.04Ti (red); (**b1**) O spectrum of T-0.1Ti (green); (**b2**) C spectrum of T-0.1Ti (red); (**c1**) O spectrum of T-0.04Ti/Nb (green); (**c2**) C spectrum of T-0.04Ti/Nb (red).

(2) Tempered specimens (Figure 13, Figure 14 and Figure 15)

Figure 13 and Figure 14 present the friction coefficients, worn surface morphologies, and cross-sectional morphologies of the tempered experimental steels. Overall, the wear of the tempered specimens is still dominated by grooves caused by abrasive action; however, the “spalling tendency” and the degree of “oxidation involvement” differ among the compositions. The wear track of T-0.04Ti exhibits relatively continuous grooves with few local cracks, and the subsurface deformation layer thickness is approximately 9.20 μm (Figure 14a,d). This indicates that the combination of tempered martensite and fine TiC precipitates effectively resists abrasive cutting while maintaining sufficient toughness to suppress crack initiation, corresponding to its lowest and most stable wear mass loss (Figure 10/Table 4). In contrast, the wear track of T-0.1Ti exhibits pronounced plastic flow, cracks, and delamination features, and the subsurface deformation layer becomes significantly thicker (Figure 14b,e). This suggests that, despite tempering, the higher Ti content leads to coarser TiC particles that act as stress concentrators, promoting subsurface crack initiation and fatigue-driven spalling. Consequently, stronger near-surface cumulative plastic deformation occurs, leading to higher wear loss.

For T-0.04Ti/Nb, more prominent accumulation of oxide products and pitting features are observed on the worn surface (Figure 14c and Figure 15c). The oxygen enrichment detected by EDS (Figure 15c) confirms the presence of oxide products. A possible explanation is that these oxide products temporarily form a discontinuous third-body layer that partially isolates the contacting surfaces, as suggested by the deceleration of wear mass loss during the 30–45 min interval (Table 4). However, the subsequent wear acceleration (45–60 min) indicates that this layer is unstable under sustained shear. While our data suggest a stage-dependent role of oxidation, direct evidence of the mechanical stability of these oxide layers is beyond the scope of this study. Therefore, we describe the mechanism as ‘oxidation-assisted wear with stage-dependent third-body involvement’ rather than a fully established ‘oxidative lubrication’ process. Combined with the “mid-stage deceleration–late-stage re-acceleration” wear kinetics observed for this specimen in Section 3.3.1, its wear mechanism can be more reasonably described as a composite process: abrasive micro-cutting as the primary mechanism, with oxide products providing partial isolation and wear reduction at certain stages, while their rupture and spalling under sustained shear lead to intensified wear at later stages.

**Figure 16:**
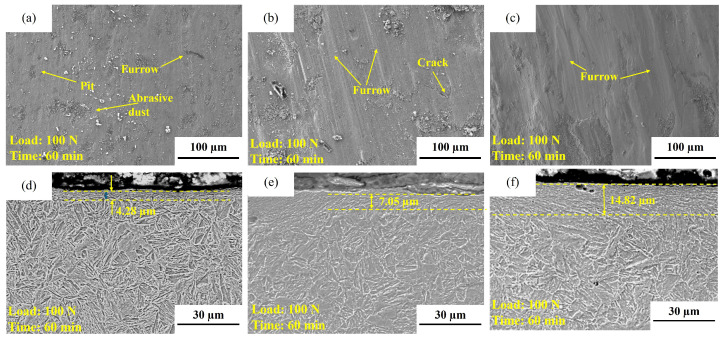
Worn surface and longitudinal cross-sectional morphologies of the experimental steel tempered at different temperatures. (**a**) Worn surface of T200-0.04Ti/Nb; (**b**) worn surface of T350-0.04Ti/Nb; (**c**) worn surface of T550-0.04Ti/Nb; (**d**) longitudinal cross-section of T200-0.04Ti/Nb; (**e**) longitudinal cross-section of T350-0.04Ti/Nb; (**f**) longitudinal cross-section of T550-0.04Ti/Nb.

(3) Effect of tempering temperature (Figure 11b and Figure 16)

To further clarify the influence of tempering-induced microstructural evolution on wear behavior, the 0.04Ti/Nb steel was selected to compare the wear responses at different tempering temperatures (Figure 11b) and the subsurface morphologies of the wear track cross sections (Figure 16). As shown in Figure 11b, under a load of 100 N for 60 min, the wear mass loss exhibits a non-monotonic dependence on tempering temperature: 71.1 ± 4.2 mg for T200-0.04Ti/Nb, increasing to 167.8 ± 1.2 mg for T350, and then decreasing to 146.6 ± 1.4 mg for T550. This result indicates that the effect of tempering temperature on wear resistance cannot be simply interpreted as “higher hardness leads to better wear resistance,” but is closely related to the material’s plastic deformation capability, crack propagation behavior, and spalling tendency during wear.

The wear track cross sections shown in Figure 16 reveal that subsurface plastic deformation and damage intensify markedly with increasing tempering temperature. The thickness of the deformation layer increases significantly from approximately 4.28 μm for T200 to about 7.05 μm for T350, and further to around 14.82 μm for T550 (Figure 16d–f). The T200 specimen exhibits a relatively thin deformation layer, and although surface grooves are clearly visible, the overall wear morphology is more stable. This suggests that, in this condition, the material possesses stronger resistance to abrasive shear deformation, and subsurface cracks are less likely to accumulate and interconnect. In contrast, the T350 specimen shows more pronounced cracking and delamination/spalling features on the worn surface (Figure 16b), corresponding to the maximum wear mass loss (Figure 11b). This indicates that contact fatigue damage is more likely to occur in this tempering state: accumulated plastic deformation at the surface promotes subsurface crack initiation and propagation, which ultimately contributes significantly to mass loss in the form of delamination spalling.

For T550, although the deformation layer is the thickest—reflecting a stronger tendency for plastic accumulation—the wear mass loss decreases compared with that of T350 (Figure 11b). Combined with the trend of increasing toughness with tempering temperature shown in Table 3, it can be inferred that enhanced toughness may, to some extent, suppress rapid crack propagation and catastrophic spalling, thereby partially offsetting the adverse effects associated with reduced strength and hardness [42]. As a result, a non-monotonic wear response characterized by “initial deterioration followed by partial improvement” is observed.

It should be noted that under the same dry-sliding conditions, oxidation may participate in the wear processes of all three tempering states. Its role is more likely manifested through the coupled effects of third-body oxide products/localized oxide layers with abrasive micro-cutting and delamination spalling at the wear interface. With increasing tempering temperature, the material surface becomes more susceptible to plastic deformation and localized spalling, and the periodic exposure of fresh metal surfaces may promote the formation of oxide products that participate in the friction interface as third bodies, thereby influencing the stage-dependent wear rate.

**Figure 17:**
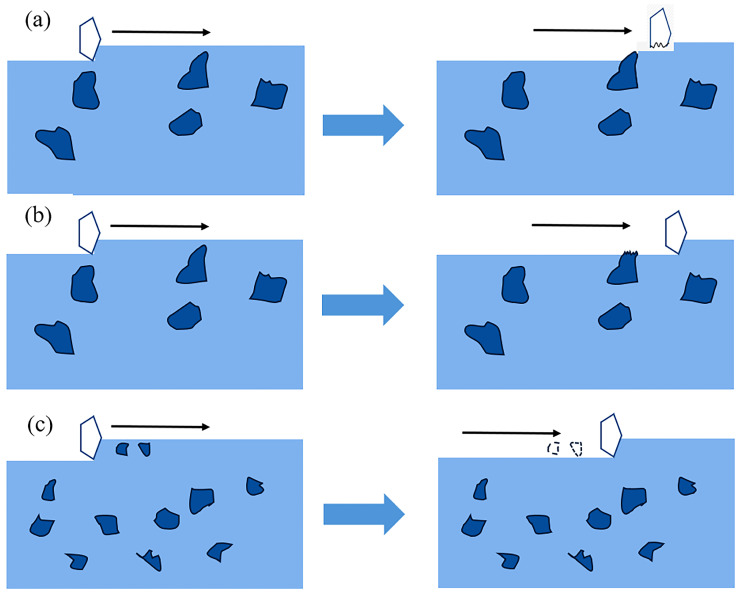
Schematic illustration of the micro-cutting wear mechanism. (**a**) Abrasive particles bypass hard particles; (**b**) hard particles are fractured; (**c**) abrasive particle size is larger than that of the hard particles.

**Figure 18:**
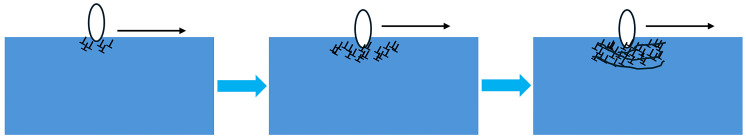
Schematic illustration of the fatigue wear mechanism.

**Figure 19:**
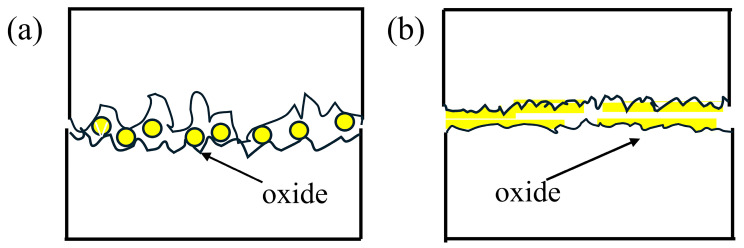
Schematic illustration of the oxidative wear mechanism. (**a**) Oxides in particulate form (yellow circles); (**b**) oxides in lamellar form (yellow rectangles).

This study presents schematic illustrations of three typical wear pathways (Figure 17, Figure 18 and Figure 19). It should be emphasized that these schematics are intended to summarize possible damage modes and diagnostic criteria rather than to replace experimental observations and quantitative results. The identification of specific mechanisms is still based on the stage-dependent evolution of wear mass loss, worn surface features, subsurface deformation morphologies, and EDS elemental distributions.

Figure 17 illustrates the abrasive micro-cutting mechanism. During sliding, abrasive particles interact with the matrix and hard carbides, resulting in continuous grooves and an approximately linear increase in wear mass loss.

Figure 18 shows the fatigue wear mechanism. Repeated shear stresses cause subsurface plastic strain accumulation, crack initiation, and eventual delamination spalling, leading to flake-like material removal and a marked increase in wear loss. This mechanism is consistent with the thickened deformation layer and enhanced spalling observed at elevated tempering temperatures.

Figure 19 depicts the possible forms of oxidation involvement. Oxide products may form as particulate debris or localized layers, temporarily isolating contacting surfaces and reducing wear. However, under sustained shear, these oxide layers fracture and spall, leading to renewed wear acceleration. Accordingly, for specimens showing oxygen enrichment and stage-dependent deceleration/re-acceleration, the wear process is described as oxidation-assisted with transient third-body effects rather than simple oxidative lubrication.

#### 3.3.3. Summary of Wear Mechanisms

By integrating the time evolution of wear mass loss (Figure 10 and Table 4) with the worn surface morphologies, subsurface deformation layers, and EDS results (Figure 12, Figure 13, Figure 14, Figure 15 and Figure 16), the wear behavior of the specimens in this study can be summarized as a composite process in which abrasive micro-cutting is the fundamental mechanism, with fatigue-induced spalling and oxide-derived third bodies acting as coupled contributors. The diagnostic criteria are as follows: continuous grooves indicate abrasive ploughing/micro-cutting; crack networks and delamination/spalling features reflect subsurface fatigue crack initiation and propagation; and oxygen enrichment in the wear track confirms oxidation involvement and suggests the possible formation of oxide-derived third bodies or localized oxide layers, whose stability governs the stage-dependent wear rate.

For the Nb-containing system, the wear kinetics of T-0.04Ti/Nb exhibits a “mid-stage deceleration–late-stage re-acceleration” behavior. Combined with oxygen enrichment in the wear track, this suggests that oxide-derived third bodies/localized oxide layers may provide stage-dependent isolation and load-bearing effects during the intermediate stage of wear; nevertheless, their rupture and spalling under sustained shear can re-intensify wear at later stages. Therefore, a more reasonable attribution is a “stage-dependent wear mitigation–failure process involving third-body oxides,” rather than a mechanism dominated solely by oxidative lubrication.

With respect to the tempering temperature effect (T200/T350/T550), the pronounced thickening of the subsurface deformation layer and the most prominent delamination/spalling at T350, consistent with its maximum wear loss, indicate that increasing tempering temperature weakens strength/hardness and promotes plastic strain accumulation and fatigue-driven spalling. Although T550 is more prone to plastic deformation, the improved toughness may suppress catastrophic delamination spalling, thereby alleviating wear loss relative to T350.

To further illustrate the relationship between material characteristics and wear performance, Table 5 summarizes the hardness, impact toughness, dominant wear mechanism, and key microstructural factor for each steel condition. It can be seen that no single mechanical property predicts wear resistance. For example, T-0.04Ti has moderate hardness (373 HB) and high toughness (133 J·cm^−2^), yielding the lowest wear loss (41.0 mg) through stable abrasive micro-cutting. In contrast, T350-0.04Ti/Nb has similar hardness (333 HB) but much lower toughness (116 J·cm^−2^), leading to severe fatigue spalling and the highest wear loss (167.8 mg). These comparisons highlight the importance of strength–toughness synergy.

The observed wear behavior can be qualitatively understood within the Archard wear framework, where wear volume is proportional to normal load and sliding distance, and inversely proportional to hardness. However, the stage-dependent kinetics and the influence of oxide-derived third bodies indicate that additional factors—such as toughness, carbide stability, and oxidation—would need to be incorporated for a more complete predictive model [43]. Developing such a model is beyond the scope of this exploratory study, but the present data provide a basis for future quantitative modelling efforts.

## 4. Conclusions

(1) Low-temperature tempering (200 °C) significantly improves the dry-sliding wear resistance of Ti-containing (0.04Ti and 0.1Ti) low-carbon martensitic steels. This improvement arises from the combination of a tempered martensitic matrix and fine TiC precipitates, which enhance resistance to abrasive shear deformation while maintaining sufficient toughness to suppress micro-cracking. The wear mass loss of T-0.04Ti and T-0.1Ti is reduced by approximately 36% and 44%, respectively, compared to their quenched counterparts under a load of 100 N for 60 min.
(2) The wear response of the Nb-containing steel (0.04Ti/Nb) cannot be described by hardness alone. It exhibits stage-dependent wear kinetics: a mid-stage deceleration (30–45 min) followed by late-stage re-acceleration (45–60 min), which correlates with oxygen enrichment on the worn surface. This behavior is attributed to the transient formation of oxide-derived third-body debris that temporarily isolates contacting surfaces, followed by their rupture and spalling under sustained shear. Thus, the wear mechanism is best described as abrasive micro-cutting with oxidation-assisted, stage-dependent third-body involvement. We note that direct identification of oxide phases and their exact role remains for future investigation using techniques such as XPS or Raman spectroscopy.
(3) The wear resistance of the 0.04Ti/Nb steel shows a non-monotonic dependence on tempering temperature: it increases from T200 to T350 and then decreases at T550. The T350 condition exhibits the highest wear loss due to pronounced subsurface plastic deformation and fatigue-driven delamination spalling. At T550, improved toughness (178 J·cm^−2^) partially suppresses catastrophic spalling, offsetting the loss in hardness. Overall, wear performance is governed by the synergistic matching of resistance to plastic deformation and resistance to crack propagation/delamination spalling, rather than by any single mechanical property. Refractory MC-type carbides (TiC/(Nb,Ti)C) act as microscale hard load-bearing units, and their interfacial stability with the matrix is a key microstructural lever for achieving the coupled strength–toughness–wear resistance design.

## Figures and Tables

**Table 1:** Chemical composition of the experimental steels.

Steel	C	Mn	Si	Al	Ti	Nb	B	N
0.04Ti	0.15	1.5	0.5	0.5	0.04	/	0.002	<0.0025
0.04Ti/Nb	0.15	1.5	0.5	0.5	0.04	0.04	0.002	<0.0025
0.1Ti	0.15	1.5	0.5	0.5	0.1	/	0.002	<0.0025

**Table 2:** Mechanical properties of the experimental steels in the quenched and tempered conditions.

Sample	Yield Strength (MPa)	Ultimate Tensile Strength (MPa)	Elongation (%)	Hardness (HB)	Impact Toughness (J·cm^−2^)
Q-0.04Ti	1030 ± 5	1421 ± 8	9.1 ± 0.2	419 ± 5	124 ± 4
Q-0.1Ti	980 ± 6	1368 ± 5	9.2 ± 0.3	402 ± 5	108 ± 5
Q-0.04Ti/Nb	970 ± 4	1322 ± 6	7.4 ± 0.4	398 ± 6	112 ± 6
T-0.04Ti	1020 ± 8	1213 ± 7	10.2 ± 0.4	373 ± 6	133 ± 4
T-0.1Ti	990 ± 5	1263 ± 4	10.3 ± 0.2	355 ± 8	128 ± 6
T-0.04Ti/Nb	1190 ± 6	1301 ± 5	10.8 ± 0.3	357 ± 5	115 ± 5

**Table 3:** Mechanical properties of the 0.04Ti/Nb experimental steel tempered at different temperatures.

Tempering Temperature (°C)	Yield Strength (MPa)	Ultimate Tensile Strength (MPa)	Elongation (%)	Hardness (HB)	Impact Toughness (J·cm^−2^)
200	1190 ± 6	1301 ± 5	10.8 ± 0.3	357 ± 5	115 ± 5
350	1035 ± 8	1252 ± 4	16.2 ± 0.6	333 ± 4	116 ± 7
550	785 ± 8	843 ± 6	23.3 ± 0.5	238 ± 3	178 ± 8

**Table 4:** Wear-test results of the experimental steels with different compositions in the quenched and tempered conditions.

Sample	15 min	30 min	45 min	60 min	Average Wear Rate (mg/min)
Q-0.04Ti	24.8 ± 4.0	41.4 ± 0.9	54.8 ± 1.8	63.8 ± 2.2	1.1
Q-0.1Ti	35.7 ± 3.7	58.2 ± 3.7	92.3 ± 3.1	128.3 ± 4.5	2.1
Q-0.04Ti/Nb	28.8 ± 6.1	45.7 ± 5.5	50.0 ± 5.5	52.2 ± 5.1	0.9
T-0.04Ti	23.4 ± 2.9	29.3 ± 3.1	33.9 ± 2.6	41.0 ± 1.5	0.7
T-0.1Ti	30.9 ± 4.8	53.1 ± 3.5	61.8 ± 5.5	72.3 ± 3.4	1.2
T-0.04Ti/Nb	19.2 ± 0.9	47.0 ± 1.7	53.5 ± 4.1	71.1 ± 4.2	1.2

**Table 5:** Mechanical properties, wear mechanisms, and microstructural factors of the experimental steels.

Specimen	Hardness (HB)	Impact Toughness (J·cm^−2^)	Dominant Wear Mechanism	Key Microstructural Factor
Q-0.04Ti	419	124	Abrasive micro-cutting	High hardness, moderate TiC
Q-0.1Ti	402	108	Micro-cutting + spalling	Coarse TiC particles
Q-0.04Ti/Nb	398	112	Micro-cutting + oxide third-body	(Nb,Ti)C, thin deformation layer
T-0.04Ti	373	133	Mild abrasive micro-cutting	Tempered martensite + fine TiC
T-0.1Ti	355	128	Micro-cutting + fatigue spalling	Tempered martensite + thick deformation layer
T-0.04Ti/Nb	357	115	Composite (micro-cutting + oxide)	Tempered martensite + (Nb,Ti)C
T350-0.04Ti/Nb	333	116	Fatigue spalling-dominated	Tempered troostite
T550-0.04Ti/Nb	238	178	Plastic deformation + mild abrasion	Tempered sorbite, high toughness

## Data Availability

The original contributions presented in this study are included in the article. Further inquiries can be directed to the corresponding author.
